# Twisted Fiber Bragg Gratings as a Polarization-Insensitive Element

**DOI:** 10.3390/s26010042

**Published:** 2025-12-20

**Authors:** Oleg V. Butov, Alexey I. Lopunov, Ivan Ulyanov, Alexey P. Bazakutsa, Alexander B. Gomzin, Egor I. Dolzhenko, Alexey B. Pnev

**Affiliations:** 1 Kotelnikov Institute of Radioengineering and Electronics of RAS, Moscow 125009, Russia; aley@mail.ru (A.I.L.); a.bazakutsa@optel.ru (A.P.B.); gomzin.ab@phystech.edu (A.B.G.); dolzhenko@phystech.edu (E.I.D.); 2 Scientific Education Center Photonics and IR Techniques, Bauman Moscow State Technical University, Moscow 105005, Russia; pniov@bmstu.ru; 3 Strooth Photonics, 33600 Pessac, France; iulyanov@strooth-photonics.com; 4 Moscow Institute of Physics and Technology, Dolgoprudny, Moscow 141701, Russia

**Keywords:** fiber bragg gratings, polarization dependence, femtosecond inscription, twisted fiber, point-by-point technique

## Abstract

This work proposes an original method for fabricating Bragg gratings using the point-by-point technique with femtosecond laser radiation, which minimizes the polarization dependence of the gratings’ optical response. The method is based on inscribing the Bragg structure in a pre-twisted optical fiber. The results of the experiments demonstrated a radical reduction in the polarization dependence of such gratings. The polarization dependence of the reflection level and Bragg wavelength in the twisted grating decreased by at least an order of magnitude and by three times, respectively, compared to the straight FBG. The results can be used in the production of high-precision Bragg sensors.

## 1. Introduction

Fiber Bragg gratings (FBGs) are one of the key groups of elements widely used in modern fiber optics, laser technology, and optoelectronics. They represent a periodic structure inscribed along the axis of an optical fiber. The periodic structure is essentially a modulation of the effective refractive index. Such gratings can serve as narrowband filters, mirrors for fiber lasers, dispersion-compensating elements, and sensing elements for physical quantities [1,2,3,4,5,6,7]. The key parameters of a Bragg grating are the reflection level (grating strength) and the reflection wavelength (Bragg wavelength). In general, the reflection level of a grating is determined by its length and the magnitude of the refractive index modulation, while the Bragg wavelength depends on the structure period and the average effective refractive index for the propagating radiation mode.

Typically, the creation of a Bragg structure (its inscription) is carried out using ultraviolet laser radiation by creating an interference pattern on the fiber core exposed to the laser beam. Permanent, non-relaxing changes in the refractive index occur at the interference maxima. Various interferometers or special phase masks, which have become the most widespread in industrial FBG manufacturing, can be used for this purpose [8,9,10,11,12]. However, this method requires the fiber waveguide to have sufficiently high photosensitivity, i.e., the ability to change the core refractive index when irradiated with UV laser light, which introduces certain complexities in the fabrication of Bragg gratings. One of the common methods to increase the photosensitivity of optical fibers for FBG inscription is hydrogen loading under high pressure [13]. However, the operational properties of such gratings, particularly their usable temperature range, are significantly limited, which, in turn, restricts their application as sensing elements under extreme conditions [14,15]. The use of molecular hydrogen in fiber laser cavity fabrication can also negatively affect its final characteristics [16]. Furthermore, the use of phase masks limits the possible range of Bragg wavelengths for the produced structures.

Alongside "classical" methods of creating FBGs with ultraviolet laser radiation, methods of direct inscription using femtosecond laser radiation have recently become widespread, particularly the point-by-point method, where each grating element (stroke) is formed by a single laser pulse [17,18,19,20,21,22,23,24]. This method involves moving the fiber at a given speed relative to the focused beam of a femtosecond laser, forming a long periodic structure (Bragg grating) pulse by pulse or “point-by-point”, hence the name of the method. The grating period is determined by the pulse repetition rate and the fiber translation speed.

A feature of this method is the impact of femtosecond radiation on the regular network of the silica glass in the fiber core, which eliminates the need for special photosensitive materials. Such methods have a number of advantages, including technological flexibility and the possibility of inscription without removing the protective polymer coating. Gratings formed by this method exhibit increased thermal stability, and the possibility of their inscription in fibers with an undoped silica core ensures high radiation resistance of Bragg sensors [25,26]. However, unlike interferometric inscription methods, where the formed grating element is a quasi-homogeneous structure in the fiber cross-section, the grating stroke formed via the point-by-point method is a filament-like defect lacking cylindrical symmetry with respect to the optical fiber axis [20,27,28,29]. Naturally, such a periodic structure will interact differently with different polarizations of radiation propagating in the fiber. This fact is confirmed experimentally. For instance, works [20,27,28,29,30,31,32] have shown a dependence of the Bragg wavelength on polarization. This effect is particularly noticeable in Bragg gratings with a phase shift in their structure. A narrow spectral dip is present in their reflection spectrum, and its position on the wavelength scale noticeably depends on the polarization of the radiation. The difference can reach several tens of picometers. Furthermore, a difference in the reflection level for different radiation polarizations is also visible [27,28,30,32]. For some applications, the difference in reflection can be beneficial, for example, for creating cavities for single-frequency fiber lasers [27,32], but such an effect negatively impacts the use of gratings as high-precision elements in optical sensors and narrowband filters. Thus, an FBG inscribed by means of the point-by-point method is a polarization-sensitive element, which in most applications adversely affects the characteristics of the final devices.

This work proposes a method to solve the problem of polarization sensitivity of FBGs inscribed by the point-by-point method using femtosecond laser radiation.

## 2. Materials and Methods

The FBG inscription was carried out using the point-by-point technology, described in detail in other works [22,23]. The experiments involved inscription in a standard Corning SMF-28 fiber (Corning, New York, NY, USA.). The inscription used a second-harmonic radiation (532 nm) from a Yb femtosecond fiber laser with a pulse repetition rate of 2 kHz. The pulse energy with a duration of 300 fs was about 80 nJ. The polarization vector of the fs laser radiation relative to the fiber axis was approximately 70 degrees. The radiation was focused with an immersion 100x microscope objective with a numerical aperture of 1.25. Each grating element (stroke) was formed by a single pulse. The fiber was moved along its axis relative to the laser beam using high-precision Aerotech ABL1000 nanopositioners (Pittsburgh, PA, USA). The translation speed was approximately 1.06 mm/s, which, considering the pulse repetition rate, defined the first-order Bragg grating with a period *Λ* of about 530 nm. The Bragg wavelength *λ_B_* was determined by Formula (1):

(1) $$\lambda_{B}=2n_{eff}\Lambda ,$$

where *n_eff_* is the effective refractive index for the radiation propagating through the grating.

**Figure 1:**
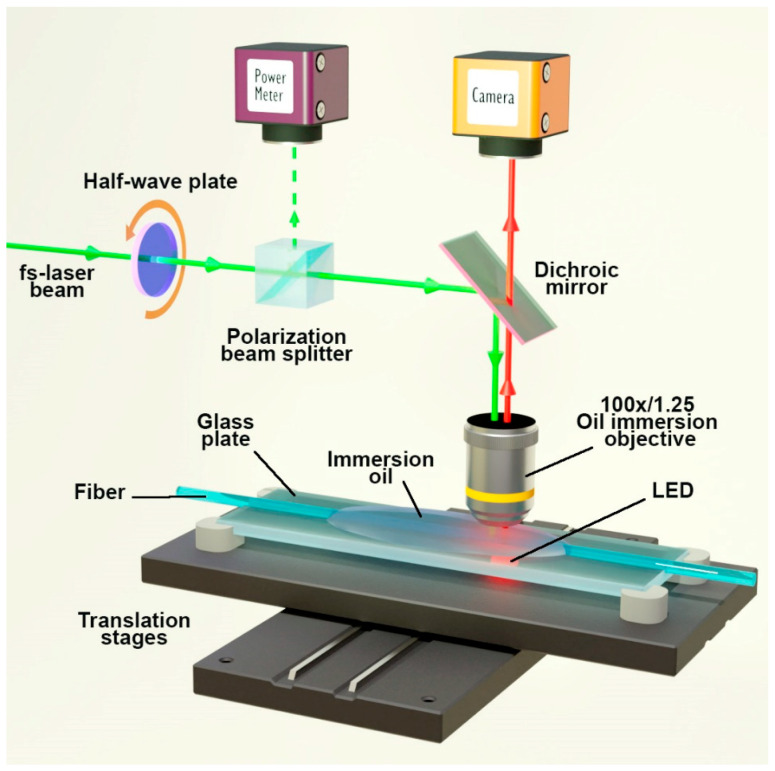
Experimental setup for fs point-by-point Bragg grating inscription.

Precise adjustment of the fiber position during inscription was ensured by an optical camera, the image of which was transmitted through a dichroic mirror via the same objective used for inscription. The inscription area was additionally illuminated from below by an LED to create a high-quality image on the camera. The setup diagram is shown in Figure 1.

**Figure 2:**
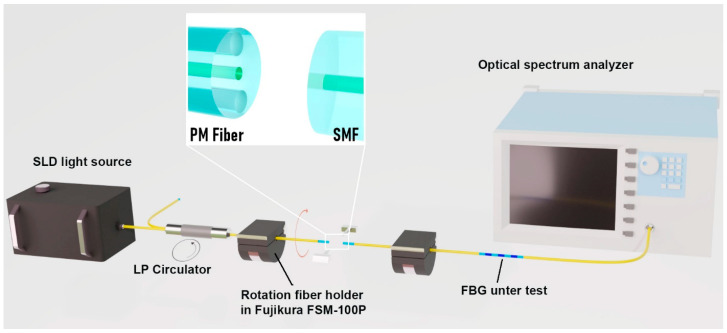
Experimental setup for analyzing the polarization dependence of the FBG response.

For analyzing the polarization dependence of the Bragg grating response, an optical scheme shown in Figure 2 was used.

Radiation from a superluminescent diode (SLD) is launched into the input port of a circulator supporting the propagation of only one polarization (LP circulator). Such a circulator was used as an efficient polarizer for our fiber circuit. The output port of the circulator, in the form of a polarization-maintaining “Panda” fiber (PM fiber), was mounted in the clamp of a Fujikura FSM-100P fusion splicer (Tokyo, Japan), which provides precision for fiber alignment and rotation of the fiber around its axis. The input end of the fiber with the inscribed Bragg grating was mounted in the second clamp of the fusion splicer. We conducted a separate experiment to control the polarization in the input fiber and confirmed that the polarization linearity does not change when the fiber is installed in the clamp. Thus, when the fibers were aligned, the launch of linearly polarized radiation into the grating was ensured, and the polarization angle of the radiation launched into the FBG was set by rotating the fiber holder. The other end of the fiber with the grating under study was connected to a Yokogawa AQ6370B optical spectrum analyzer (Tokyo, Japan). The grating strength (its reflection magnitude) and the Bragg wavelength were determined from the measured transmission spectrum. The setup allowed the measurement of the dependence of these key grating parameters on the rotation angle of the launched radiation polarization.

The relationship between the reflection coefficient *R* and the grating strength $\kappa_{B}$ is given by the Formula (2):

(2) $$R={tanh}^{2}\left(\kappa_{B}L\right),$$

where *L* is the grating length. Naturally, the grating strength $\kappa_{B}$ is determined by the average magnitude of the refractive index modulation created by the laser-induced defects *Δn_mod_* [4]:

(3) $$\kappa_{B}=\frac{\pi \Delta n_{mod}\eta }{\lambda_{B}},$$

where *η* is the so-called overlap integral, which in our case determines the fraction of the energy of the radiation passing through the grating that crosses the cross-section of the defect. Under identical grating inscription conditions, this parameter can be considered constant.

For the convenience of analyzing experimental data, we will use the total grating strength *κ_B_L*.

## 3. Results and Discussion

**Figure 3:**
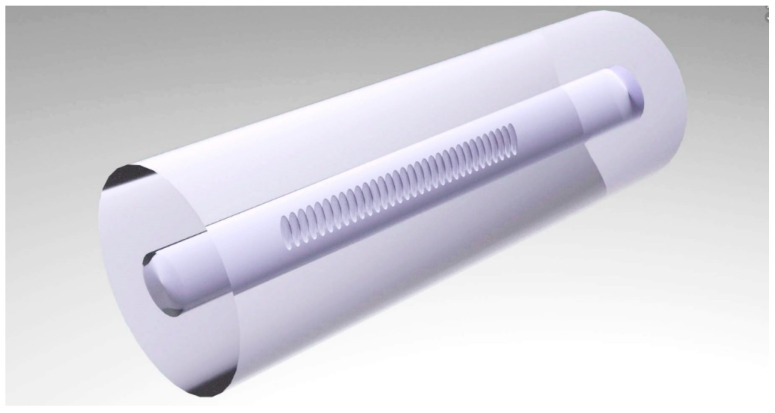
Structure of a fiber Bragg grating inscribed by the point-by-point method using focused femtosecond laser radiation.

As mentioned in the introduction, each element of a grating inscribed by the point-by-point method represents a filament-like defect lacking cylindrical symmetry in the optical fiber cross-section (Figure 3).

**Figure 4:**
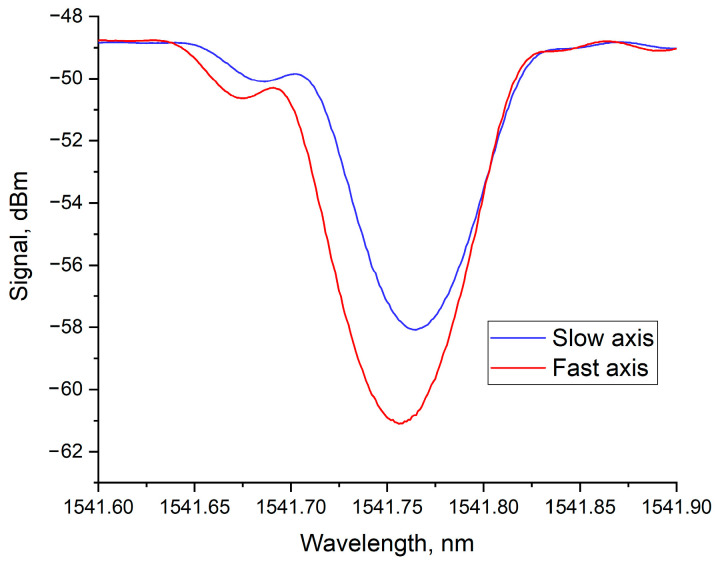
Transmission spectra of the FBG for mutually orthogonal polarizations of the transmitted radiation.

This defect shape arises from the specifics of its formation in the region of the laser radiation focus. In the transverse cross-section, the intensity distribution in the focused laser beam is close to a Gaussian distribution with an effective width approaching the diffraction limit. The intensity distribution along the vertical direction in the focal region (i.e., the intensity distribution of the radiation along the depth relative to the central axis of the laser beam) has a more extended structure and depends on the objective aperture. Furthermore, near the focal point, additional effects of radiation self-focusing can be observed, which may increase the effective interaction length with the material along the beam axis [33,34]. Thus, the formed defect also has an elongated structure, extending along one of the axes of the optical fiber cross-section. Naturally, a periodic structure formed from such asymmetric defects interacts differently with radiation of different polarizations, which is reflected in the transmission spectrum of the Bragg grating as a dependence on the angle of the polarization vector of the radiation propagating in the fiber. The transmission spectra of the same Bragg grating, measured for mutually orthogonal polarizations, are shown in Figure 4.

The graph shows that the grating strength (its reflection or the “depth” of the spectrum) differs for different polarizations of the transmitted radiation. Moreover, the Bragg reflection wavelength also differs, indicating a difference in the average effective refractive index.

A higher reflection level indicates a larger magnitude of refractive index modulation, which can occur when the polarization vector of the radiation coincides with the direction of the filament axis in the grating structure. In this case, the value of the overlap integral *η* in Formula (3) is larger. It should be noted that the spectrum of the grating with the higher reflection level is shifted towards the shorter wavelength region, indicating a lower effective refractive index compared to the orthogonal polarization, where the influence of the filament-like grating structure on reflection is smaller. This effect can manifest itself when there is a negative change in the refractive index in the main structure of the formed defect, which fully correlates with the results of another study [24]. By analogy with polarization-maintaining fiber, we will call the “fast axis” of the grating the axis where the radiation polarization coincides with the filament axis, and the axis orthogonal to it will be called the “slow axis”.

**Figure 5:**
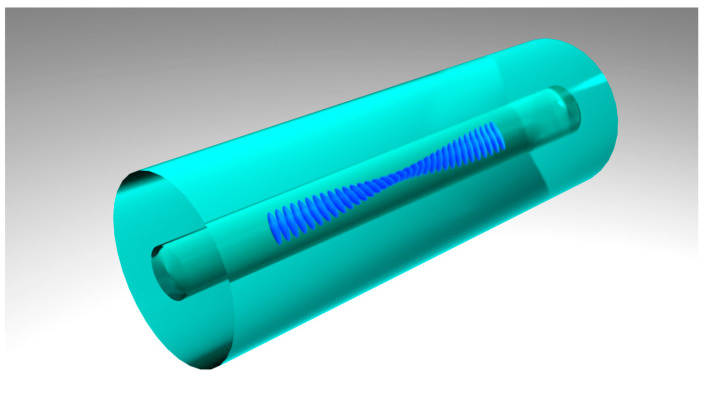
Schematic representation of the twisted Bragg structure.

To solve the problem of polarization dependence of FBGs inscribed by the point-by-point method, we propose creating a Bragg grating with a helical (twisted) structure, in which the direction of each subsequent filament-like grating element is located at an angle to the previous one in the plane of the optical fiber cross-section (Figure 5). Thus, a rotation of the entire structure along its length by π (or a total angle multiple of π) should lead to the averaging of the birefringence effect, ensuring polarization independence of the Bragg grating response.

During standard FBG inscription, the fiber is placed in a holder groove and fixed with clamps on both sides. For the creation of the proposed structure, the optical fiber is twisted along its axis before being mounted in the holder. The inscription is then carried out by the usual method, leading to the formation of a straight-line structure, as shown in Figure 3, but within the twisted fiber. After removal from the clamps, the fiber returns to its original state, forming the twisted structure of the Bragg grating itself (Figure 5). The twist period is chosen so that the full length of the grating corresponds to an integer number of half-turns, ensuring the condition mentioned in the previous paragraph.

**Figure 6:**
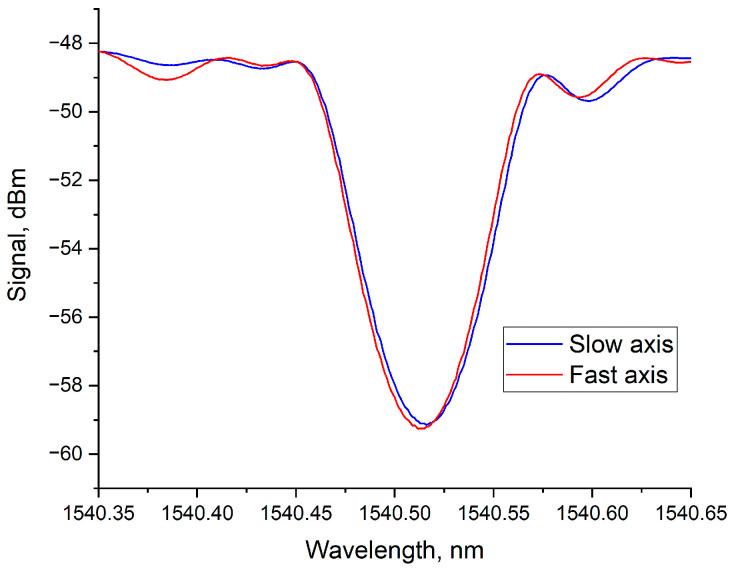
Transmission spectra of the twisted FBG for mutually orthogonal polarizations of the transmitted radiation.

In our experiment, the grating length (*L*) was 15 mm, equaling one full turn of the twist (2π). The grating inscription parameters were identical to those used for inscribing the straight FBG whose spectrum is shown in Figure 4. The transmission spectra of the new twisted Bragg grating for mutually orthogonal polarizations are presented in Figure 6.

As can be seen from the graphs in Figure 4 and Figure 6, the grating with a straight structure retains strong polarization dependence. The transmission dip for radiation propagating along the fast axis of the grating is more than 12 dB, while for the slow axis, this value is around 9 dB. Furthermore, the Bragg wavelengths differ by about 10 pm. In the case of the grating inscribed under the same conditions but in a pre-twisted fiber (twisted FBG), practically no difference in the reflection level is observed, and the absolute value of the transmission dip is slightly less than 11 dB. The difference in Bragg wavelengths also decreased significantly and is no more than 3 pm. It should be noted that this difference might be observed due to minor errors in positioning the inscribed grating relative to the fiber center.

**Figure 7:**
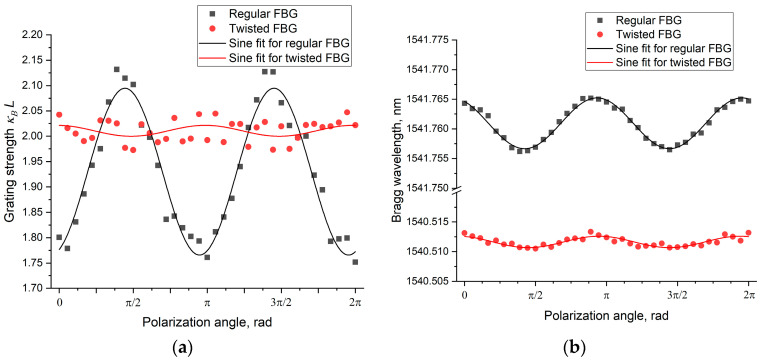
Dependence of the grating strength *κ_B_L* (**a**) and the Bragg wavelength (**b**) on the rotation angle of the polarization vector of the radiation propagating in the fiber for FBGs inscribed by the standard method (black) and in twisted fibers (red).

For a more detailed analysis of the effect in these gratings, measurements were carried out at various polarization angles with an angular step of 10 degrees (π/18). The experimental results are presented in the graph in Figure 7 as the dependence of the total grating strength (*κ_B_L*) (Figure 7a) and the Bragg wavelength (Figure 7b) on the angle of the polarization vector of the radiation propagating in the fiber. Experimental data are shown as points on the graph. The results were fitted by a sinusoidal function, which corresponds to the nature of the observed dependencies. The result of the fitting of the experimental data is shown on the graph as solid lines. As can be seen from the graphs, for the twisted FBG, this dependence is about an order of magnitude smaller for the grating strength and three times smaller for the Bragg wavelength compared to the grating inscribed in a straight, non-twisted fiber. The results of the experiments testify to the effectiveness of the proposed FBG inscription method for radically reducing the polarization dependence of their parameters, which is especially important in the fabrication of high-precision Bragg sensors.

## 4. Conclusions

The parameters of fiber Bragg gratings inscribed by the point-by-point method using femtosecond laser radiation, such as the reflection level and Bragg wavelength, are sensitive to the polarization of the radiation propagating in the fiber due to the cylindrical asymmetry of the fs laser pulse-induced grating elements (strokes) in the optical fiber cross-section, which represent a filament-like structure. To minimize such polarization dependence, this work proposes a method for creating a helical grating structure (twisted FBG). Such a structure can be created by conventional linear grating inscription in a pre-twisted optical fiber. The conducted comparative analysis of the spectra of the inscribed FBGs demonstrated the effectiveness of the proposed method. The polarization dependence of the reflection level (grating strength) and Bragg wavelength in the twisted grating decreased by at least an order of magnitude and by three times, respectively, compared to the straight FBG. This technique can be used for the fabrication of high-precision Bragg sensors that are insensitive to the polarization of the radiation used to read their parameters.

## Data Availability

Data are contained within the article.
